# Supplementary material for: Superior colliculus peri-saccadic field potentials are dominated by a visual sensory preference for the upper visual field
Source: iScience. 2025 Feb 13;28(3):112021. doi: 10.1016/j.isci.2025.112021 (PMC11914513; doi:10.1016/j.isci.2025.112021)
Supplement: Document S1. Figures S1–S3 [file mmc1.pdf]

iScience, Volume *28*

Supplemental information

Superior colliculus peri-saccadic field potentials are dominated by a visual sensory preference for the upper visual field

Ziad M. Hafed

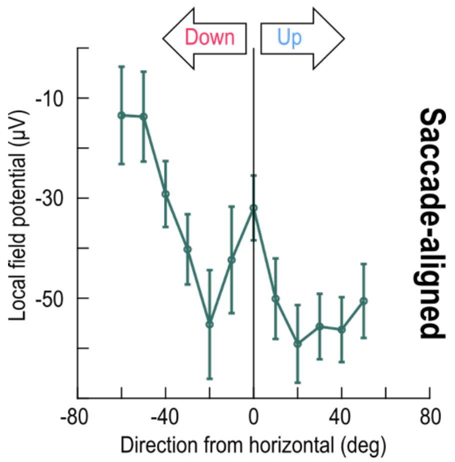

Figure S1. Stronger pre-saccadic local field potential (LFP) negativity in the upper visual field representation of the primate superior colliculus (SC), related to Fig. 1. This figure shows an analysis similar to that of Fig. 1F, except that I now measured the LFP in the final 50 ms before saccade onset, instead of peri-saccadically. There was still a stronger negativity in the upper visual field representation of the SC. Error bars denote SEM. Statistically, I grouped all upper visual field electrode tracks into one group and all lower visual field electrode tracks into another, and I then performed a t-test during the pre-saccadic interval (Methods). The result revealed a significant effect ($p=0.0022$; $t=-3.0985$; $n=95$ and 102 for upper and lower visual field tracks, respectively), and that upper visual field pre-saccadic LFP negativity was ~1.6 times larger than lower visual field LFP negativity (in absolute value). In addition, a one-way ANOVA across the shown angular direction bins revealed a significant effect of direction from the horizontal meridian ($p=0.0006$; $F(11,339)=3.05$; $n=351$). Thus, the results of Fig. 1D-F also held in the pre-saccadic interval when the peri-saccadic LFP modulation was still just building up.

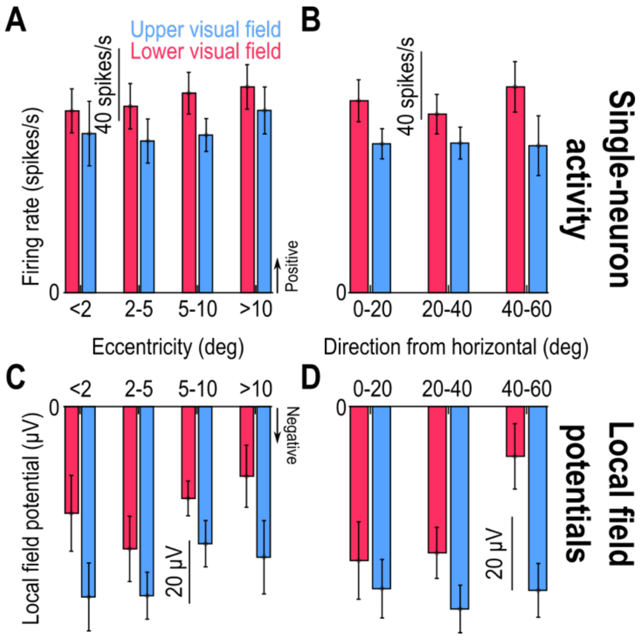

Figure S2. Consistency of the results of Fig. 2 in the pre-saccadic interval, related to Fig. 2. I repeated the analyses of Fig. 2, but now taking measurements during the final 50 ms before saccade onset. There were similar results to those seen in Fig. 2. Note how the LFP asymmetry was generally stronger than the spiking asymmetry (compare blue and red bars in each eccentricity or direction bin). Statistically, I compared the LFP measurements in **C** using a two-way ANOVA with the two main factors of the ANOVA being visual field location (upper or lower visual field) and eccentricity represented by the electrode track location penetrating the SC. I obtained a significant main effect for upper/lower visual field location ($p=0.0086$; $F(1,185)=7.04$; $n=193$) but not for eccentricity ($p=0.1892$; $F(3,185)=1.61$; $n=193$). Similarly, for **D**, I performed a two-way ANOVA with the main factors of the ANOVA being upper/lower visual field location and direction from the horizon. Again, there was a significant main effect of upper/lower visual field location ($p=0.0087$; $F(1,166)=7.05$; $n=172$) but not direction ($p=0.2389$; $F(2,166)=1.44$; $n=172$). For the spiking activity, there was a marginal main effect of upper/lower visual field location ($p=0.0994$; $F(1,185)=2.74$; $n=193$) but not eccentricity ($p=0.7266$; $F(3,185)=0.44$; $n=193$) in **A**; in **B**, there was a significant main effect of upper/lower visual field location ($p=0.0119$; $F(1,166)=6.47$; $n=172$) but not direction ($p=0.8579$; $F(2,166)=0.15$; $n=172$). Thus, in both the spiking and LFP's, there were consistent asymmetries between the upper and lower visual field electrode track locations during pre-saccadic epochs. Error bars denote SEM.

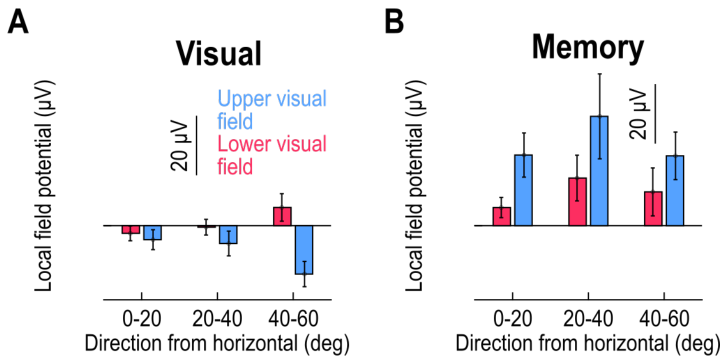

Figure S3. Stronger delay-period LFP negativity in the upper visual field during visual stimulation, and stronger delay-period LFP positivity in the upper visual field during working memory, related to Fig. 3. (A) I measured the LFP value at the end of the delay period in the visually-guided saccade task (from -50 ms to +25 ms relative to the saccade "go" signal; Methods). Across all sites, there was stronger LFP negativity in the upper, rather than lower, visual field (Fig. 3). I then binned the electrode sites according to which direction from horizontal was represented by the recorded SC activity (similar to Figs. 2, 4, 6, S2). For all upper visual field direction bins, the LFP value at the go signal was more negative than for the lower visual field direction bins. This was confirmed statistically using a two-way ANOVA (main factors being upper/lower visual field location and direction from the horizon): there was a main effect of upper/lower visual field location ($p=0.0018$; $F(1,166)=10.08$; $n=172$) but not of direction bin ($p=0.9038$; $F(2,166)=0.1$; $n=172$). Thus, there was stronger LFP negativity in the SC's upper, rather than lower, visual field representation (Fig. 1A-C), even during sustained visual stimulation conditions (beyond the initial sensory-evoked transients). **(B)** In the memory-guided version of the saccade task, there was no visual stimulus driving the recorded neurons at the time of the go signal. In this case, the LFP amplitude during the delay period became positive for all electrode sites (Fig. 3), but there was still a strong upper/lower visual field effect: across direction bins, there was stronger LFP positivity for the upper visual field sites than for the lower visual field ones ($p=0.0193$ for the main effect of upper/lower visual field location with $F(1,103)=5.65$, $n=109$; and $p=0.3112$ for the main effect of direction bin, with $F(2,103)=1.18$, $n=109$; two-way ANOVA). Thus, working memory altered LFP values in the SC (compare **A** to **B**), but there was still a clear difference between the upper and lower visual field SC representations, even in the absence of a visual stimulus. Error bars denote SEM.
